# South West Orthopoedic Club

**Published:** 1986-08

**Authors:** 


### South West Orthopoedic Club

*Abstracts of papers given at the meeting in Bristol November, 1983.*

### PRIDIE MEMORIAL LECTURE

**'Modern Trends in the Management of Brachial Plexus Lesions'**

**Dr. C. B. Wynn Parry, Director of Rehabilitation, Royal National Orthopaedic Hospital**

Dr. Wynn Parry gave an excellent Pridie Memorial Lecture based on his large experience of patients with brachial plexus injuries. He described the types of injury, the mechanism of the injury and the clinical presentations. He discussed the surgery, stressing the need for early exploration, but concentrated on the management by presenting much he had developed both in the RAF and later at the Royal National Orthopaedic Hospital. The lecture was appreciated by a large audience of members of the South West Orthopaedic Club and some members of the Physiotherapy staff.

He was introduced by Professor Brian Pickering, Professor of Anatomy at the University of Bristol and Dean of the Medical School.

### MALIGNANCY AND SILASTIC PARTICLES

**Mr I. Winson (Bristol)**

This paper, presented by Mr. Winson, described the results of a study of the carcinogenic properties of silastic particles placed intra-articularly in an animal model. 50 rats had had silastic particles placed in a knee and a further 50 rats had been similarly treated with high density polyethylene particles as controls. Following sacrifice at 21 months, there had been no increase in the incidence of carcinoma in either group. It had been noted, however, that though the HDP particles had stayed locally in the knee, the silastic appeared to have cleared from the knee.

It had therefore been concluded that in this animal model intra-articular particulate silastic did not have a carcinogenic potential but Mr. Winson felt that further studies of the dynamics of silastic particles would be justified.

### HEMIARTHROPLASTY OF THE HIP

**Mr. A. Baker (Bristol)**

Mr. Baker reported on a retrospective review of 233 hemiarthroplasties which had been performed. All the results had been evaluated clinically and radiologically. Overall the clinical results had been poor with 56% complaining of hip pain, 34% being limited by their hip and only 11% having normal function.

### THE USE OF BONE SCANNING IN PAINFUL CONDITIONS OF THE FEET

**Mr. H. Maurice (Bristol)**

This paper discussed the usefulness of bone scans of the feet. 76 patients had been reviewed and the reason for the foot scan and the part played by the scan in reaching the diagnosis was discussed. The conclusion was that bone scanning of the feet was useful to confirm bone or joint infection, to diagnose soft tissue injury, to diagnose lesions poorly shown on plain radiographs i.e. stress fractures, fractured sesamoids, subtalar arthritis, some bone tumours and tarsal coalitions and to investigate the painful foot when other investigations were normal.

### A REVIEW OF THE EARLY EXPERIENCES OF THE METHOD AND RESULTS OF 38 SHOULDER ARTHROSCOPIES

**Mr. T. D. Bunker (Nottingham)**

A series of 38 shoulder arthroscopies were presented, and the history, surgical anatomy and portal sites were demonstrated. The method of shoulder arthroscopy was demonstrated and the normal structures as seen at arthroscopy were discussed. Abnormalities of the long head of biceps, the rotator cuff, glenoid labirum, glenoid, humerus and ligaments were shown as well as loose bodies, needling and probling.

Mr. Bunker discussed the results of surgery, problems encountered and complications and a review of world literature was presented.

Mr. Bunker concluded by saying that shoulder arthroscopy had been found to be a useful diagnostic tool in the differential diagnosis of shoulder pain, a useful research tool for the shoulder surgeon and promised to be an effective therapeutic agent for synovial biopsy, removal of loose bodies, cuff debridement, repair of recurrent dislocations, but, however, for the present it must remain a research tool for the shoulder surgeon until the limitations were better defined.

### LATE POSITIONAL CORRECTION OF UNITING FEMORAL FRACTURES USING THE EXTERNAL FIXATOR

**Mr. C. H. Marsh (Bath)**

Mr Marsh described the use of the Wagner leg lengthening external fixator in six patients with extensive femoral shaft fractures. Good fracture fixation was combined with the facility for distraction and lengthening of the early reparative callus at up to 10 weeks post injury. The method was recommended for thos patients where fracture shortening and malalignment had persisted as union proceeded.

### THE DIAGNOSTIC SPECTRUM OF AVASCULAR NECROSIS

**Professor L. Solomon (Bristol)**

Professor Solomon reported that although bone death had long been recognised as a complication of osteomyelitis and certain fractures, avascular necrosis in the absence of injury had been seen with increasing frequency during the last two decades. Among the common causes today, were high-dosage corticosteroid therapy and alcohol abuse. In both of these there had been an abnormality of lipid metabolism with progressive accumulation of fat in the marrow and a marked rise in intra-medullary pressure. It had been posited that this could lead to sinusoidal occlusion and vascular stasis with resulting marrow ischaemia. A common factor in all types of bone death was ischaemia or, more precisely, a disparity between the oxygen need of the bone cell and the ability of the local circulation to supply that need. This could be brought about by four types of disorder.

Professor Solomon went on to report that in Type I

AVN there was arterial cut-off (e.g. after a fracture). In Type II AVN there is intravascular arteriolar occlusion (e.g. in sickle-cell disease). In Type III AVN it was posited that there may be major venous occlusion and in Type IV AVN there was sinusoidal compression (e.g. in Gaucher's disease and in steroid-induced AVN). In the latter group there was the possibility of reversing the process at an early stage by surgical decompression of the cancellous bone end.

**HAGLUND'S SYNDROME**

**Mr. S. Young (Bristol)**

Fifty-two cases of Haglund's Syndrome seen consecutively over a six year period have been reviewed. Thirty-nine patients with sixty affected feet were available for follow up. Eighteen cases involving twenty-eight feet had been treated conservatively and twenty-one cases involving thirty-two feet had been treated surgically. Eighty-two per cent of those treated conservatively and seventy-two per cent of those treated surgically were asymptomatic at follow up. There was a high rate of surgical complication. A review of the literature is presented and the value of radiology in this condition discussed.

We suggest that patients who present with this condition should initially be treated conservatively, with attention to shoe wear and that x-rays are unhelpful. It is better to make the shoe fit the foot rather than alter the foot to fit the shoe.

